# Auxin-Induced Adventitious Rooting in Pepper Involves *CaLBD16*: Functional Evidence from Tomato Overexpression

**DOI:** 10.3390/plants15081188

**Published:** 2026-04-13

**Authors:** Xinhao Zhang, Bingqian Tang, Hongyan Shen, Kai Li, Min He, Qianqian Du, Zhiyi Yin, Lin Xie, Meiqi Wang, Manman Yang, Jiayue Li, Zhuo Zhang, Feng Liu

**Affiliations:** 1 Engineering Research Center for Germplasm Innovation and New Varieties Breeding of Horticultural Crops, Key Laboratory for Vegetable Biology of Hunan Province, College of Horticulture, Hunan Agricultural University, Changsha 410125, China; 17267298729@163.com (X.Z.); bqtang@hunau.edu.cn (B.T.); calson0202@163.com (K.L.); he_min2001@163.com (M.H.); 13607473835@163.com (Q.D.); yinzy@stu.hunau.edu.cn (Z.Y.); 4040@stu.hunau.edu.cn (L.X.); meiqiwanghnau@126.com (M.W.); 18196390777@163.com (M.Y.); jiayueli@stu.hunau.edu.cn (J.L.); 2 Vegetable Variety Creation Center, Yue Lushan Lab, Changsha 410128, China; 3 School of Tropical Agriculture and Forestry, Hainan University, Haikou 570228, China; 17863808135@163.com; 4 Microbial Variety Creation Center of Yuelushan Laboratory, Yue Lushan Lab, Changsha 410128, China; 5 Institute of Plant Protection, Hunan Academy of Agricultural Sciences, Changsha 410125, China

**Keywords:** pepper, *CaLBD16*, auxin, adventitious root

## Abstract

The formation of adventitious roots plays a key role in plant asexual reproduction, yet research on this process in pepper remains limited. Exogenous auxin treatment substantially promotes adventitious root formation in pepper, and we identified *CaLBD16* as a positive regulator acting downstream of auxin signaling. Further analysis revealed that the signal transduction from auxin to *CaLBD16* is mediated through the activation of CaARF6 and CaWOX11, both of which also contribute to adventitious root formation. Together, our findings uncover a molecular mechanism underlying auxin-induced adventitious root formation in pepper, offering valuable insights for its practical application in production.

## 1. Introduction

Adventitious roots (AR) arise from non-root tissues, and their formation is a critical step in cutting propagation [1]. Through clonal propagation via cuttings, it is possible to multiply such as insect or disease resistance. Multiple hormones influence AR formation, including auxin, ethylene, jasmonic acid, and cytokinins [2,3,4,5], indicating that this process is complex. Among these, endogenous auxin plays the most critical role and is widely used in tissue culture to induce rooting [6]. At the physiological level, IAA gradually accumulates at wound sites, thereby promoting AR formation [7]. Treatments with exogenous IAA can stimulate the differentiation of phloem cells, creating a way for unidirectional endogenous auxin canalization, contributing to the initiation of AR primordia [4]. At the molecular level, auxin biosynthesis, transport, and signaling are closely linked to AR formation through the activation of key genes. These include auxin biosynthesis genes such as *AtYUC2* and *AtYUC6*; auxin transporters like *AUX1*, *LAX3* [8], and *PIN1* [9]; and auxin signaling components, including auxin response factors such as *ARF4*, *ARF6*/*8*, and *ARF7* [10,11,12]. In addition, auxin/indole-3-acetic acid (Aux/IAA) family members—including *IAA4* [13], *IAA14* [14], *IAA13*, and *IAA16* [15]—have been demonstrated to participate in the regulation of AR formation.

AR formation involves a shift in cell fate, a process in which the WOX gene family plays a key role. *WOX11* regulates the first step—from regeneration-competent cells to root founder cells—while *WOX5*, activated downstream, controls the second step, driving the transition from root founder cells to AR primordium [10,16]. In Arabidopsis, the expression of both *AtWOX11* and *AtWOX5* during AR formation is induced by auxin [16]. In addition, members of the LBD gene family have been shown to be essential for AR formation. In particular, *LBD16*/*29*, *LBD18*, and *SBRL* have been functionally implicated in this process across different species, among them, *LBD16* and *LBD29* are considered to be direct downstream targets of *WOX11* [4,17,18].

Pepper (*Capsicum annuum*) is a globally important vegetable crop, yet many elite cultivars exhibit poor adventitious rooting capacity, which limits their efficient propagation via cuttings. Although auxin is well known to promote adventitious root formation in various plant species, its role and the underlying molecular mechanisms in pepper remain entirely unexplored. Given the woody characteristics of pepper stems and the lack of research on its de novo root organogenesis, it is essential to identify key regulatory factors that mediate auxin-induced adventitious rooting in this crop. Elucidating such mechanisms will not only advance our understanding of root development in pepper but also provide a theoretical basis for improving cutting propagation efficiency in commercial production.

In this study, we found that *CaLBD16* plays a key role in de novo root organogenesis. Notably, exogenous auxin treatment significantly promoted AR formation in pepper. We further demonstrated that *CaARF6* mediates auxin signaling to activate *CaLBD16* expression. Beyond this canonical auxin signaling module, *CaWOX11* was also found to respond to auxin and activate *CaLBD16* transcription. Collectively, these findings suggest that the regulatory framework governing AR formation is relatively conserved across plant species and provide a theoretical basis for improving AR formation in pepper.

## 2. Results

### 2.1. Exogenous IAA Significantly Promotes AR Formation in Pepper

**Figure 1:**
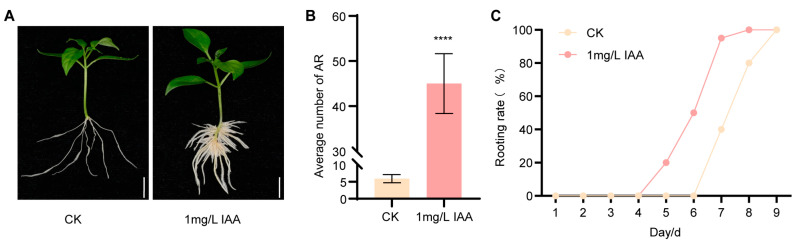
Exogenous IAA treatment promotes adventitious root (AR) formation in pepper. (**A**) Morphological observation of two-week-old pepper cuttings (with roots removed) cultured on MS medium for 10 days with 1 mg/L exogenous IAA treatment, using control medium as a control. (**B**) Counting of adventitious root (AR) numbers within 10 days of exogenous IAA treatment. (**C**) Statistical analysis of the AR formation rate. Data are means ± SE [*n* = 15 (**A**,**B**)], *n* represents the biological replicate. Differences (**** *p* < 0.0001) are based on two-tailed Student’s *t*-test.

To investigate AR formation in pepper, we selected cultivar S8—a widely grown yet difficult-to-root line—as the experimental material. Two-week-old tissue-cultured seedlings were excised and transferred to either basal MS medium or MS medium supplemented with 1 mg/L IAA, and phenotypic observations were recorded over 10 days. After 10 days of treatment, explants cultured on IAA-containing medium produced approximately eight times the number of ARs as the control (Figure 1A,B). Moreover, AR emergence was observed by day 5 under IAA treatment, whereas in the control group, ARs did not appear until day 7 (Figure 1C).

### 2.2. Overexpression of CaLBD16 Significantly Promotes AR Formation in Tomato

**Figure 2:**
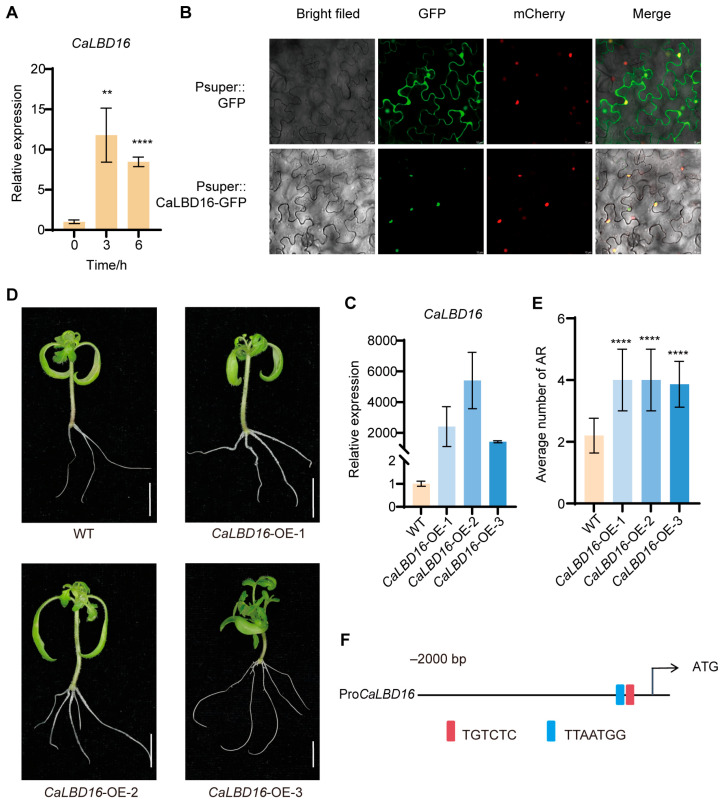
*CaLBD16* mediates IAA signaling to promote AR formation. (**A**) *CaLBD16* was upregulated in response to IAA treatment. (**B**) CaLBD16-GFP is localized to the nucleus in differentiated tobacco cells, along with the nuclear marker AtHY5-mCherry. Scale bar = 10 μm. (**C**) Three tomato lines overexpressing *CaLBD16* were identified by qRT-PCR. (**D**) Morphological observation of AR formation in *CaLBD16*-overexpressing tomato cuttings compared with WT. (**E**) Quantification of AR formation in (**D**). (**F**) Promoter analysis of CaLBD16. Data are means ± SE [*n* = 3 (**A**,**C**), *n* = 15 (**D**,**E**)], *n* represents the biological replicate. Differences (** *p* < 0.01, **** *p* < 0.0001) are based on two-tailed Student’s *t*-test.

Hypocotyls of cultivar S8 were treated with 1 mg/L IAA and collected for qRT-PCR analysis to examine the expression of *CaLBD16*, a key transcription factor known to promote AR formation in multiple species [4,17,19]. The results showed that *CaLBD16* expression was significantly upregulated in response to exogenous IAA treatment (Figure 2A). Subcellular localization analysis further revealed that CaLBD16 localizes to the nucleus (Figure 2B), consistent with its anticipated role as a transcription factor. The *CaLBD16* gene was cloned and introduced into tomato for overexpression, resulting in three independent transgenic lines (Figure 2C). Two-week-old tissue-cultured seedlings of these lines were subjected to cutting propagation, and AR numbers were subsequently recorded. Compared with the wild type (WT), all three *CaLBD16*-overexpression lines exhibited significantly enhanced AR formation, with an average of approximately 4 ARs per explant compared to approximately 2 in the WT (Figure 2D,E), suggesting that the role of *CaLBD16* in promoting AR formation is evolutionarily conserved across plant species.

Given that *CaLBD16* expression is auxin-responsive, the molecular mechanism underlying its induction by auxin was further explored. Promoter analysis of *CaLBD16* revealed the presence of two conserved cis-regulatory elements upstream of the ATG start codon: a canonical ARF-binding motif (TGTCTC) at position −190 bp and a WOX11-binding motif (TTAATGG) at −194 bp (Figure 2F). These findings provide important clues for identifying upstream regulators involved in the auxin-mediated transcriptional control of *CaLBD16*.

### 2.3. CaARF6 Activates CaLBD16

**Figure 3:**
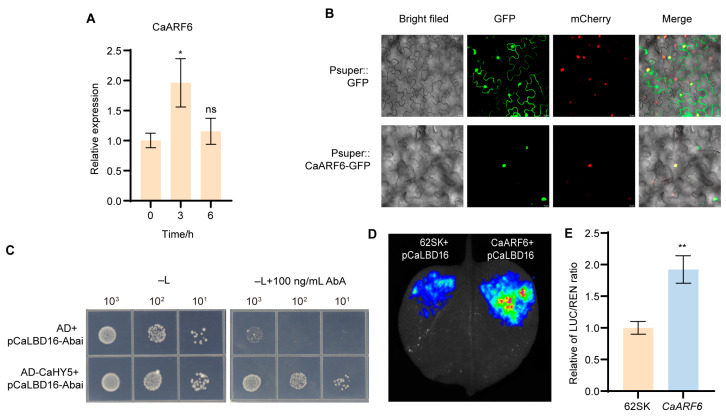
CaARF6 activates *CaLBD16* expression in response to IAA signaling. (**A**) *CaARF6* was upregulated in response to IAA treatment. (**B**) CaARF6-GFP is localized to the nucleus in differentiated tobacco cells, along with the nuclear marker AtHY5-mCherry. Scale bar = 10 μm. (**C**) Yeast one-hybrid assays demonstrated that CaARF6 binds to the *CaLBD16* promoter fragment. (**D**,**E**) Dual-LUC reporter gene assay demonstrating transactivation of *CaLBD16* by CaARF6 in *N. benthamiana* leaves. The activity of firefly LUC and REN was assessed by co-expression of 35S:*CaARF6* with Pro*CaLBD16*:LUC in *N. benthamiana* leaves. The empty vector served as the control. Data are means ± SE [*n* = 3 (**A**,**E**)], *n* represents the biological replicate. Differences (ns *p* > 0.05, * *p* < 0.05, ** *p* < 0.01,) are based on two-tailed Student’s *t*-test.

ARF transcription factors are known to play important roles in AR formation. To investigate whether *CaARF6* in pepper responds to auxin and participates in this process, we first examined its expression under IAA treatment. The results showed that *CaARF6* exhibited an expression pattern similar to that of *CaLBD16*, with significant upregulation in response to auxin (Figure 3A). Subcellular localization analysis revealed that CaARF6 localizes to the nucleus (Figure 3B), consistent with its role as a transcription factor.

To determine whether CaARF6 directly binds the TGTCTC element, tandem repeats of this motif were used as bait in a yeast one-hybrid (Y1H) assay. The results confirmed that CaARF6 specifically binds to this element (Figure 3C). Using an artificial differentiated system, transient overexpression dual-luciferase reporter assays in *Nicotiana benthamiana* leaves further demonstrated that CaARF6 significantly activates *CaLBD16* expression, with an approximately 1.9-fold increase (Figure 3D,E).

### 2.4. Overexpression of CaARF6 Significantly Promotes AR Formation in Tomato

**Figure 4:**
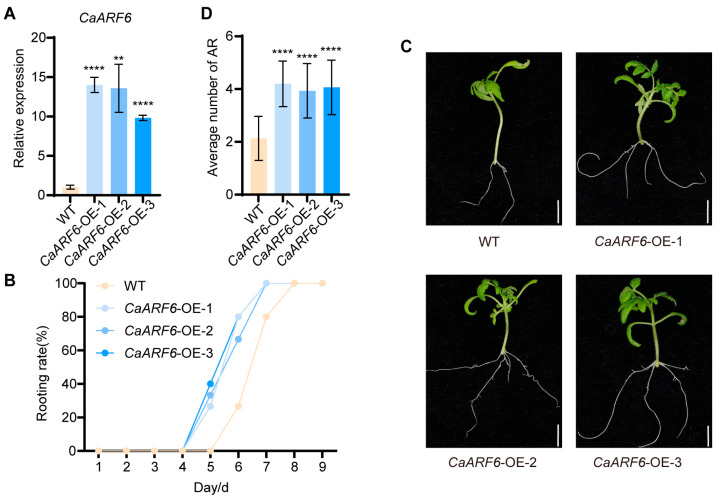
CaARF6 promotes AR formation. (**A**) Three tomato lines overexpressing CaARF6 were identified by qRT-PCR. (**B**) Rooting rates of CaARF6-overexpressing tomato cuttings within 9 days compared to WT. (**C**) Morphological observation of AR formation in CaARF6-overexpressing tomato cuttings compared with WT. (**D**) Quantification of AR formation in B. Data are means ± SE [*n* = 3 (**A**), *n* = 15 (**B**–**D**)], *n* represents the biological replicate. Differences (** *p* < 0.01, **** *p* < 0.0001) are based on two-tailed Student’s *t*-test.

The 35S:CaARF6 construct was introduced into tomato, and three independent transgenic lines were successfully obtained (Figure 4A). Two-week-old hypocotyls of *CaARF6*-OE lines were excised at the base and transferred to fresh MS medium for AR induction. Root emergence was observed in *CaARF6-*OE lines at 5d, whereas in WT plants, ARs did not appear until 6d (Figure 4B). Subsequent quantitative analysis revealed that the CaARF6-OE lines produced significantly more ARs than the WT, with an average of approximately four roots per plant compared to only about two in the WT (Figure 4C,D). These results indicate that *CaARF6* promotes AR formation.

### 2.5. CaWOX11 Activates CaLBD16

**Figure 5:**
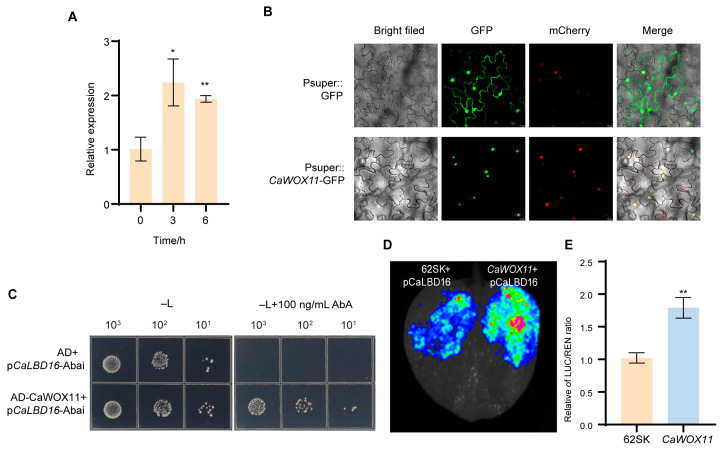
CaWOX11 activates *CaLBD16* expression in response to IAA signaling. (**A**) *CaWOX11* was upregulated in response to IAA treatment. (**B**) CaWOX11-GFP is localized to the nucleus in differentiated tobacco cells, along with the nuclear marker AtHY5-mCherry. Scale bar = 10. (**C**) Y1H assays demonstrated that CaWOX11 binds to the *CaLBD16* promoter fragment. (**D**,**E**) Dual-LUC reporter gene assay demonstrating transactivation of *CaLBD16* by CaWOX11 in *N. benthamiana* leaves. The activity of firefly LUC and REN was assessed by co-expression of 35S: *CaWOX11* with Pro*CaLBD16*:LUC in *N. benthamiana* leaves. The empty vector served as the control. Data are means ± SE [*n* = 3 (**A**,**E**)], *n* represents the biological replicate. Differences (* *p* < 0.05, ** *p* < 0.01,) are based on two-tailed Student’s *t*-test.

Given that the *CaLBD16* promoter contains a TTAATGG motif identical to the WOX11-binding element reported in apple [4], we investigated whether CaWOX11 regulates *CaLBD16* in pepper. First, expression analysis showed that *CaWOX11* was significantly upregulated in hypocotyls in response to IAA treatment (Figure 5A), consistent with the expression pattern of *CaLBD16* (Figure 2A). Transient overexpression of Psuper:*CaWOX11* in *Nicotiana benthamiana* leaves revealed that CaWOX11 localizes to the nucleus (Figure 5B), consistent with its function as a transcription factor.

To determine whether CaWOX11 directly binds the TTAATGG element, tandem repeats of this motif were used as bait in a Y1H assay. The results demonstrated that CaWOX11 specifically binds to this element (Figure 5C). Using an artificial differentiated system, transient overexpression dual-luciferase reporter assays in *Nicotiana benthamiana* leaves further demonstrated that CaWOX11 significantly activates *CaLBD16* expression, with an approximately 1.7-fold increase (Figure 5D,E).

### 2.6. Overexpression of CaWOX11 Significantly Promotes AR Formation in Tomato

**Figure 6:**
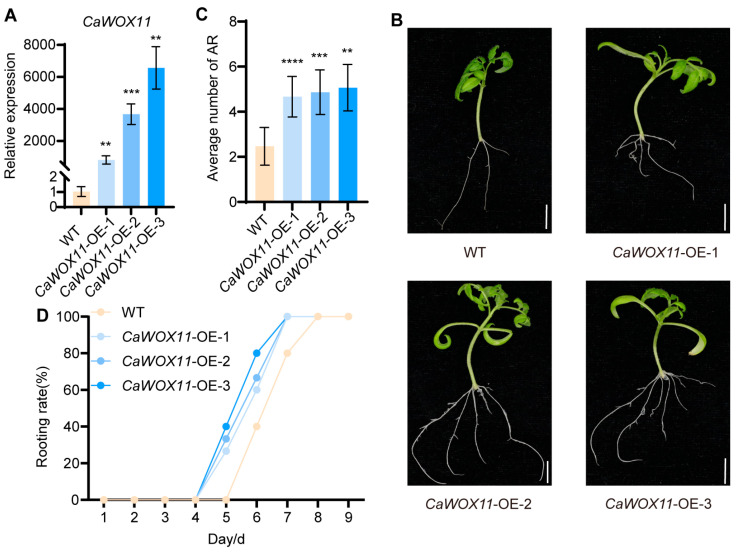
*CaWOX11* promotes AR formation. (**A**) Three tomato lines overexpressing *CaWOX11* were identified by qRT-PCR. (**B**) Morphological observation of AR formation in *CaWOX11*-overexpressing tomato cuttings compared with WT. (**C**) Quantification of AR formation in (**B**). (**D**) Rooting rates of *CaWOX11*-overexpressing tomato cuttings within 9 days compared to WT in (**B**). Data are means ± SE [*n* = 3 (**A**), *n* = 15 (**B**–**D**)], *n* represents the biological replicate. Differences (** *p* < 0.01, *** *p* < 0.001, **** *p* < 0.0001) are based on two-tailed Student’s *t*-test.

The *CaWOX11* gene was overexpressed in tomato, and three independent transgenic lines were successfully generated (Figure 6A). Hypocotyls of two-week-old tissue-cultured seedlings from *CaWOX11*-OE lines were excised at the base and transferred to fresh MS medium. AR number and rooting rate were recorded over a nine-day period. The results showed that CaWOX11-OE lines exhibited more than twice the number of ARs compared with the WT (Figure 6B,C) and accelerated AR emergence (Figure 6D).

## 3. Discussion

AR formation is a crucial step in asexual propagation, which serves as an important means of multiplying horticultural crops. Considerable variation in adventitious rooting capacity exists among different pepper cultivars, highlighting the significance of understanding the underlying molecular mechanisms in this species. The formation of ARs occurs as a result of the polar transport of auxin, which establishes an auxin gradient and subsequently leads to the differentiation of root primordium founder cells under auxin stimulation. Exogenous auxin can only mimic endogenous auxin by providing a transient pulse effect. This study investigated the role of exogenous auxin treatment in simulating the process by which auxin promotes adventitious root formation in peppers, and elucidated the connection between the auxin signal transduction pathway and AR formation [20].

Accumulating evidence has demonstrated that auxin promotes adventitious root formation across a wide range of plant species, including *Arabidopsis* [21], water fern [22], *Ulmus pumila* [3], tomato [23], sweet potato [24], and *cucumber* [25]. In line with these findings, our experiments in pepper also showed that auxin enhances AR formation during cutting propagation (Figure 1). This suggests that the promoting role of auxin in AR formation is likely conserved across the plant kingdom.

LBD transcription factors have been implicated in the development of various plant organs, including lateral roots [17], flowers [26], and leaves [27]. Several members of this family are also known to regulate AR formation, such as *LBD16*/*29* [28], *LBD17* [18], and *LBD18* [17]. Among these, *LBD16* has been reported to control adventitious rooting in multiple species, including *Arabidopsis*, peach [29], poplar [30], and maize [31]. However, whether *LBD16* plays a similar role in pepper remained unknown. In this study, we overexpressed *CaLBD16* in tomato and observed a significant promotion of AR formation. Moreover, *CaLBD16* expression was upregulated in response to IAA treatment, suggesting that IAA induces *CaLBD16* to enhance adventitious rooting in pepper (Figure 2).

The signaling pathway through which auxin regulates *CaLBD16* in pepper warrants further investigation. ARF transcription factors, as key mediators of auxin signaling, have been implicated in AR formation across diverse species. Notably, studies indicate that ARFs ultimately converge on the activation of *LBD16* to exert their function, as observed in peach [29] and poplar [30]. In this study, we identified a canonical ARF-binding motif (TGTCTC) within the *CaLBD16* promoter and experimentally validated *CaARF6* as a direct upstream regulator (Figure 3). Furthermore, phenotypic analysis confirmed that *CaARF6* promotes AR formation (Figure 4).

De novo AR formation requires cell fate transition, a process in which *WOX11* and *WOX5* have been shown to play key roles [16]. These factors subsequently promote adventitious rooting through the activation of *LBD16* [32]. In apple and *Arabidopsis*, the WOX11-binding element has been identified as TTAATGG [4,10]. Promoter analysis of *CaLBD16* revealed the presence of this element at position −194 bp relative to the start codon. Experimental validation confirmed that CaWOX11 regulates *CaLBD16* expression in pepper. Notably, compared with the element positions reported in apple and Arabidopsis, this motif is located closer to the start codon in pepper, suggesting a potentially stronger regulatory effect.

Although auxin is widely recognized for its role in promoting AR formation, its function in pepper has not been specifically investigated. Our study demonstrates that exogenous IAA serves as a key hormone in promoting adventitious rooting in pepper. Furthermore, we elucidated a regulatory pathway—conserved in other species—by which mechanical wounding induces AR formation through auxin activation, mediated by *CaWOX11* and *CaARF6*, with *CaLBD16* at its core. These findings provide a theoretical basis for improving rooting efficiency in pepper production and offer valuable insights for breeding cultivars more amenable to cutting propagation.

## 4. Materials and Methods

### 4.1. Plant Materials and Treatment

Seeds of pepper S8 were surface-sterilized and sown on 1/2 MS medium (Coolaber, Beijing, China). Two weeks later, the basal parts of the hypocotyls were excised and transferred to MS medium supplemented with 1 mg/L IAA (Sigma-Aldrich, St. Louis, MO, USA) for subculture. Tomato seeds were similarly sterilized and germinated on1/2 MS medium supplemented with 1.5% (*w*/*v*) sucrose and solidified with 0.8% agar. After two weeks, the basal hypocotyl segments were cut and subcultured onto MS medium (Coolaber, Beijing, China) supplemented with 3% (*w*/*v*) sucrose and solidified with 0.8% agar containing 1 mg/L IAA as well as onto hormone-free MS medium. All plant materials were maintained in sterile growth chambers under controlled conditions: a day/night temperature of 26/23 °C, a 16 h light/8 h dark photoperiod, and a light intensity of 144 μmol/m^2^/s.

S8 seedlings at two weeks of age were treated by spraying the hypocotyls with a solution containing 1 mg/L IAA and 0.1% (*v*/*v*) Triton X-100 (Solarbio, Beijing, China). Samples were harvested from hypocotyls at 0, 3, and 6 h after treatment, immediately flash-frozen in liquid nitrogen, and kept at −80 °C until RNA isolation. Each biological replicate consisted of pooled tissue from five individual plants, and three independent replicates were collected per time point.

### 4.2. Construction of Transgenic Overexpression

The effects of overexpressing *CaARF6*, *CaWOX11*, and *CaLBD16* under the control of the 35S promoter were investigated in *Ailsa Craig* (AC). Full-length coding sequences of these genes were amplified from pepper S8 and cloned into the pHellgate 8 vector. The constructs were then heterologously introduced into tomato plants via Agrobacterium rhizocarpus-mediated transformation with strain GV3101. Transgenic plants were confirmed by PCR analysis, and transgene expression levels were determined by RT-qPCR.

### 4.3. RNA Isolation and Expression Analysis

Total RNA was isolated from frozen tissue samples using the SteadyPure Plant RNA Extraction Kit. Approximately 1 μg of RNA was reverse-transcribed into cDNA with the HiScript II 1st Strand cDNA Synthesis Kit (Vazyme, Nanjing, China). Quantitative real-time PCR (qRT-PCR) was performed on a Roche LightCycler^®^ 480 system (Roche Diagnostics, Basel, Switzerland) using ChamQ Universal SYBR qPCR Master Mix (Vazyme, Nanjing, China) in 96-well plates. The housekeeping gene (Caz06g27840/Solyc11g005330.1) served as an internal reference [33], and all primer sequences are provided in Table S1.

### 4.4. Subcellular Localization

The coding sequences of *CaARF6*, *CaWOX11*, and *CaLBD16* (excluding stop codons) were inserted into the 35S::pCAMBIA1300-GFP vector. The resulting constructs and the empty vector were introduced into *Nicotiana benthamiana* leaves via *Agrobacterium rhizogenes* strain GV3101, along with the nuclear marker *AtHY5*-mCherry. Fluorescence was visualized using a Zeiss LSM510 META confocal microscope (Carl Zeiss, Oberkochen, Germany) with excitation/emission wavelengths of 488/510 nm for GFP and 561/610 nm for mCherry.

### 4.5. Yeast One-Hybrid Assay

The full-length coding sequences of *CaARF6* and *CaWOX11* were inserted into the pGADT7 vector. A tandem triple repeat of the *CaLBD16* promoter fragment (AAGCCATTAATAA or AAATGTCTCCTA) was synthesized and cloned into the pAbAi bait vector following BstBI (New England Biolabs, Ipswich, MA, USA) digestion. The resulting construct was linearized and integrated into the yeast Y1H Gold (WeiDi Biotechnology, Shanghai, China) genome to generate reporter strains. These strains were subsequently transformed with either AD-*CaARF6* or AD-*CaWOX11*. Transformants were selected on SD/-Leu/-Ura medium (Coolaber, Beijing, China) at 28 °C for 72 h. Positive colonies were resuspended in distilled water to an OD600 of 0.2, and aliquots were spotted onto SD/-Leu/-Ura plates with or without aureobasidin A (AbA) (Coolaber, Beijing, China). Yeast cells co-transformed with empty pGADT7 and pAbAi-*CaLBD16* served as the negative control.

### 4.6. Dual-Luciferase Transactivation Assay

For binding activity assays, a 2000 bp promoter fragment upstream of the start codon of each target gene was cloned into the pGreen II 0800-LUC vector as the reporter. This vector also contains a 35S-driven Renilla luciferase (REN) cassette serving as an internal control. The full-length coding sequences of *CaARF6* and *CaWOX11* were inserted into the pGreen II 62-SK vector to generate effector constructs, while the empty 62-SK vector was used as a negative control. All constructs were transformed into *Agrobacterium tumefaciens* GV3101. Appropriate combinations of agrobacterial cultures were mixed and co-infiltrated into *Nicotiana benthamiana* leaves. After three days, the leaves were sprayed with luciferin and images were captured using a CCD camera (e.g., NEWTON 7.0 Bio) (Vilber, Marne-la-Vallée, France). Firefly and Renilla luciferase activities were quantified using a dual-luciferase assay kit (Promega, Madison, WI, USA).

## Data Availability

The data used to support the findings of this study are available upon request to the corresponding author.

## Supplementary Materials

The following supporting information can be downloaded at: https://www.mdpi.com/article/10.3390/plants15081188/s1, Table S1: Primers used in the present study.
