# Supplementary material for: Auxin-Induced Adventitious Rooting in Pepper Involves CaLBD16: Functional Evidence from Tomato Overexpression
Source: Plants (Basel). 2026 Apr 13;15(8):1188. doi: 10.3390/plants15081188 (PMC13120054; doi:10.3390/plants15081188)
Supplement: Supplementary file 1 [file plants-15-01188-s001.zip › plants-4210499-supplementary.pdf]

Table S1 All primers used in this study

Prime name	Prime sequence (5'- 3')
**Primers used for Y1H assays**	
CaWOX11-AD-F	GTACCAGATTACGCTCATATGATGGAAGACTCTACCTCGTCAA
CaWOX11-AD-R	ATGCCCACCCGGGTGGAATTCTTACTTCCTCCCTTCCTGTGCA
CaARF6-AD-F	GTACCAGATTACGCTCATATGATGAGGGTATCTTCAGCTGG
CaARF6-AD-R	ATGCCCACCCGGGTGGAATTCCTACTTGTAGTTTAGGACGATGTT
CaLBD16-pAbAi-F	AAAATGATGAATTGAAAAGCTTCCTCTTCGATACTCTGGTCTC
CaLBD16-pAbAi-R	ACAGAGCACATGCCTCGAGGCTTCCAGGATGTATTTGTTAA
**Primers used for Dual-luciferase transactivation assay**	
CaARF6-62SK-F	GCCGCTCTAGAACTAGTGGATCCATGAGGGTATCTTCAGCTGG
CaARF6-62SK-R	TTGGTACCGGGCCCCCCCTCGAGTTAGTAGTCAAGTGACCCCAATG
CaWOX11-62SK-F	GCCGCTCTAGAACTAGTGGATCCATGGAAGACTCTACCTCGTCAA
CaWOX11-62SK-R	TTGGTACCGGGCCCCCCCTCGAGCTACTTGTAGTTTAGGACGATGTT
CaLBD16-0800-F	CACTATAGGGCGAATTGGGTACCCCTCTTCGATACTCTGGTCTC
CaLBD16-0800-R	TATGTTTTTGGCGTCTTCCATGGGATAGAAGTGCACACGAGCA
**Primers used for construction of transgenic overexpression**	
CaWOX11-OE-F	CATTTGGAGAGGACACGCTCGAGATGGAAGACTCTACCTCGTCAA
CaWOX11-OE-R	TCTCATTAAAGCAGGACTCTAGACTTGTAGTTTAGGACGATGTTTGC
CaARF6-OE-F	CATTTGGAGAGGACACGCTCGAGATGAGGGTATCTTCAGCTGG
CaARF6-OE-R	TCTCATTAAAGCAGGACTCTAGAGTAGTCAAGTGACCCCAATGGTATCC
CaLBD16-OE-F	CATTTGGAGAGGACACGCTCGAGATGGCTTCTGGTACAGGGT
CaLBD16-OE-R	TCTCATTAAAGCAGGACTCTAGAGCTTTTCATCATCCTAAGAGCC
**Primers used for qRT-PCR**	
CaWOX11-qRT-F	ATGGGTCTTCCGCAAGTTGA
CaWOX11-qRT-R	GGACGATGTTTGCACAGATGA
CaARF6-qRT-F	TCACAAGTACAGCGGGCAAT
CaARF6-qRT-R	TTGGTCCCCATTTTCGGAGG
CaLBD16-qRT-F	GCCTGATAGGTGTGAGGCTG
CaLBD16-qRT-R	AGCTGAGTCTTCGCTTGCAT
CaUBI-qRT-F	TGTCCATCTGCTCTCTGTTG
CaUBI-qRT-F	CACCCCAAGCACAATAAGAC
SlActin-qRT-F	GGAGATTGAAACTGCCAGGAGCA
SlActin-qRT-R	CTGCAGCTTCCATACCAATCATGG
